# SELDI蛋白质指纹对因基因多态性导致耐药性漂移诊断的前瞻性研究——吉非替尼与含铂方案序贯治疗非小细胞肺癌

**DOI:** 10.3779/j.issn.1009-3419.2013.01.06

**Published:** 2013-01-20

**Authors:** 利娜 吉, 毅 裴

**Affiliations:** 030032 太原，山西医学科学院，山西大医院老年肿瘤科 Department of Geriatric Medical Oncology, Shan Xi Da Yi Hospital, Taiyuan 030032

**Keywords:** SELDI技术, 化疗, 吉非替尼, 肺肿瘤, SELDI, Chemotherapy, Gefitinib, Lung neoplasms

## Abstract

**背景与目的:**

本研究组前期研究发现借助于SELDI技术可以预测吉非替尼的疗效，而且指出其蛋白质指纹图谱M/Z：8, 693±50H^+^丰度≤25%，可以作为吉非替尼治疗非小细胞肺癌（non-small cell lung cancer, NSCLC）患者取、舍的筛选指标，以这个指标为界定，观察含铂方案治疗失败的患者以蛋白质指纹为依据指导化疗与吉非替尼的序贯应用的方法的远期效果。

**方法:**

选择TP、DP和GP方案化疗失败，且经SELDI检测M/Z（质核比）8, 693±50H^+^的丰度≤15%的NSCLC患者9例，口服吉非替尼250 mg，1次/d，治疗期间每2个月查患者血清SELDI指纹，以M/Z: 8, 693±50H^+^的丰度 > 25%作为再化疗指标，并以M/Z: 8, 693±50H^+^丰度≤15%为继续服用吉非替尼的指标，两者之间根据肿块变化和肿瘤标记物变化决定取舍吉非替尼治疗时间，指导吉非替尼与含铂方案的序贯治疗，观察总生存时间。

**结果:**

随访至2010年12月，9例患者中位总生存期为27个月（10个月-66个月）。

**结论:**

SELDI技术通过指纹的描述，有计划地选择这些抗肿瘤药物治疗NSCLC的取、舍，可提高药物治疗的获益率和获益时间。

## Abstract (en)

**Background and objective:**

This study aims to observe the long-term effects of sequential therapy with a platinum-based regimen and gefitinib for non-small cell lung carcinoma (NSCLC) with failed chemotherapy under the guidance of surface-enhanced laser desorption/ionization (SELDI).

**Methods:**

Nine NSCLC patients with failed chemotherapy and ≤15% abundance of M/Z: 8, 693±50H^+^ were selected. The patients were administered with 250 mg of gefitinib (p.o., once a day). During the therapy, SELDI was applied every 2 months. If the M/Z: 8, 693±50H^+^ abundance was > 25%, chemotherapy was applied; if the abundance was ≤15%, gefitinib (p.o.) was applied. In between, we chose gefitinib according to the mass and tumor markers. On the base, we sequentially applied the drug and observed the total survival time.

**Results:**

Follow-ups until December 2010 revealed that the median overall survival time of the nine patients was 27 months (10 months-66 months).

**Conclusion:**

The treatment benefit ratio and benefit time of anti-NSCLC drugs can be improved by SELDI fingerprinting.

恶性肿瘤抗肿瘤治疗失败多因耐药所致，包括原发性耐药和继发性耐药两种，多表现为一种多药耐药的特点。耐药的出现必须换药治疗已成共识，但是再换的药又发生耐药该如何处理呢？对此，我们课题小组采用2008年发现的SELDI蛋白质组指纹可以筛选出吉非替尼治疗非小细胞肺癌（non-small cell lung cancer, NSCLC）的优势人群和耐药发生这一研究结果^[1]^，设计对含铂方案化疗失败后选择吉非替尼（gefitinib）治疗的患者根据SELDI蛋白质指纹M/Z: 8, 693±50H^+^的丰度为选择含铂方案化疗和继续服用吉非替尼治疗的依据，有计划地进行化疗和吉非替尼的序贯治疗，现将观察结果报告如下。

## 1 资料与方法

### 1.1 病例资料

本组9例患者为2006年12月-2009年10月我科收治的病理诊断明确的NSCLC，男性1例，女性8例。年龄37岁-76岁，中位年龄56岁。病理学类型：腺癌8例（均为女性），肺泡细胞癌1例（为男性），入组患者在服用吉非替尼前均接受过至少2个周期以上的TP（紫杉醇+顺铂）、DP（多西他赛+顺铂）或GP（吉西他滨+顺铂）方案化疗，影像学检查提示肿瘤增大或有转移，根据指纹M/Z：8, 693±50H^+^丰度≤15%，服用吉非替尼治疗，动态观察其指纹丰度，> 25%停用吉非替尼治疗并至少接受1次以上TP、DP或GP方案化疗后，两者之间根据肿块变化和肿瘤标记物变化决定取舍吉非替尼治疗时间，又接受吉非替尼治疗并有效或稳定者，完成序贯周期交替达1次以上直至死亡。其中肺内转移1例，骨转移2例，肝转移2例，脑转移1例。

### 1.2 实验工作

#### 1.2.1 主要仪器、软件及试剂

PBS-IIc表面增强飞行时间质谱仪（SELDI-TOF-MS）、能量吸收分子EMA、CM10型蛋白质芯片及相应分析软件，HFPFS缓冲盐（Sigma公司），CHAPS缓冲盐（Sigma公司）。

#### 1.2.2 标本采集及处理

首次治疗前取清晨空腹末梢静脉血3 mL，静置，3, 000 r/min离心5 min分离血清，-80 ℃冰箱保存。血清标本冰浴解冻，10, 000 r/min离心2 min。取5 μL样品，加10 μL 9 M尿素。振荡30 min，加180 μL缓冲液稀释（100 mmol/L NaOAc pH 4.0），4 ℃ 10, 000 r/min离心2 min，取上清待用。

#### 1.2.3 实验方法

① 芯片预处理：将芯片安装到Bioprocessor上，每孔加200 μL结合缓冲液（100 mmol/L NaOAc pH4.0），振荡5 min，拍干，重复1次。每孔上样100 μL血清样品，室温振荡1 h。甩去液体，用200 μL结合缓冲液洗2次，每次5 min，拆下芯片，待芯片自然干燥，然后每点加SPA（乙腈50%和三氟乙酸0.5%的饱和溶液）0.5 μL两次，干燥待测。②数据收集：用加有All-in-one标准蛋白质的NP20芯片校正质谱仪，设定仪器参数，在Ciphergen ProteinChip软件中设定读片程序读取芯片数据，其中横坐标为蛋白质质荷比（M/Z），纵坐标为蛋白质相对含量（丰度）。

### 1.3 序贯治疗方法

#### 1.3.1 化疗方案

选择TP（紫杉醇+顺铂）、DP（多西他赛+顺铂）或GP（吉西他滨+顺铂）方案。

#### 1.3.2 序贯治疗路径

经TP、DP和GP方案化疗失败的患者，通过SELDI检测，若M/Z（质核比）8, 693±50H^+^的丰度≤15%，接受口服吉非替尼250 mg，1次/d，治疗期间每2个月查患者血清SELDI指纹，以M/Z: 8, 693±50H^+^的丰度 > 25%作为再选择含铂方案化疗指标，并以M/Z: 8, 693±50H^+^丰度≤15%为继续服用吉非替尼的指标，两者之间根据肿块变化和肿瘤标记物变化决定取舍吉非替尼治疗时间，指导吉非替尼与含铂方案的序贯治疗（图 1）。

**1:**
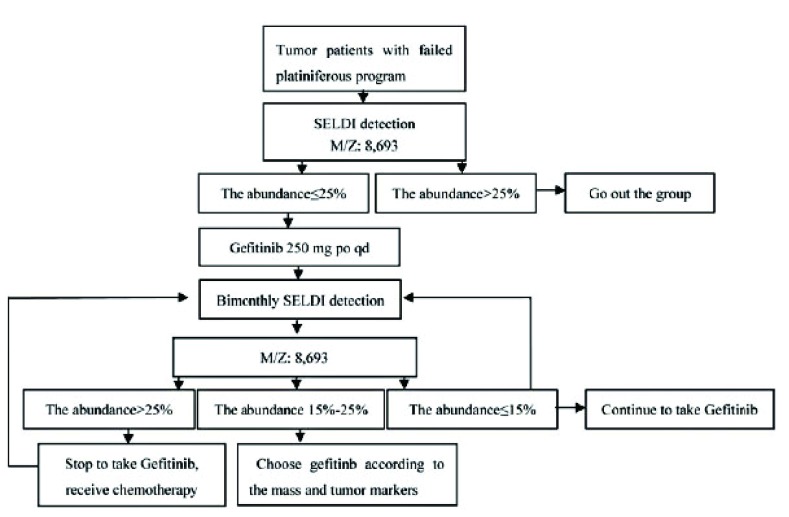
吉非替尼与含铂方案序贯治疗非小细胞肺癌治疗路径 The sequential therapy of non-small cell lung cancer (NSCLC) with platinum-based regime and Gefitinib

### 1.4 疗效评价标准

肿瘤病灶疗效评价标准采用WHO实体肿瘤疗效评价标准^[2]^，包括完全缓解（complete response, CR）、部分缓解（partial response, PR）、疾病稳定（stable disease, SD）和疾病进展（progresive disease, PD）。

### 1.5 研究观察标准

对所有患者每2个月随访一次，直至死亡，观察总生存期。

## 2 结果

随访至2010年12月（最后一个患者去世），9例患者中位总生存期为27个月（10个月-66个月）。9例患者中，有4例规律行化疗及吉非替尼序贯治疗，2例患者服吉非替尼期间出现肝转移，行化疗，疗效SD后继续口服吉非替尼，另3例患者因为化疗副作用或经济原因未严格完成序贯周期。

## 3 讨论

含铂方案化疗是目前治疗NSCLC的主要化疗方法，一旦出现耐药，再选择治疗是很困难的，靶向药物酪氨酸激酶受体抑制剂（tyrosine kinase inhibitor, TKI）吉非替尼的出现，无疑为这些患者带来希望，一系列的临床研究都在探索吉非替尼一线治疗NSCLC的多种模式及其可能性，然而研究^[3, 4]^表明吉非替尼与含铂方案同步联合一线治疗晚期NSCLC，与单纯一线化疗相比未能显示出生存优势，可能解释为TKI与细胞毒化疗药物之间有潜在的拮抗作用。为克服TKI与化疗同步联合所带来的拮抗效应，专家们设计靶向药物与化疗药物的序贯联合，以表皮生长因子受体（epidermal growth factor receptor, EGFR）表达作选择靶向药物的取舍标记，也未得出有意义的结论^[5]^。在BR.21和ISEL两大多中心随机临床试验中^[6-8]^，也发现吉非替尼单药用于晚期耐药性NSCLC并不能明显改善生存期。这是由于约80%的肺癌患者服用EGFR-TKI11个月左右出现耐药复发，丧失对其原有的敏感性^[9-11]^，其依然存在有耐药问题，因此化疗与EGFR-TKI联合应用有望加强NSCLC的治疗效果。

现有的理论认为肿瘤是个异质体，这与肿瘤基因的多态性有关^[12-13]^，目前针对*EGFR*的基因分层也发现同种NSCLC瘤体内EGFR分布有差异，这些差异均表明对吉非替尼治疗的敏感性不同^[14]^。因此面对含铂方案化疗耐药并吉非替尼再治疗失败的患者，我们设想其原因是否与肿瘤异质体有关，形成一种特殊的无效治疗现象：即敏感肿瘤被杀灭后不敏感肿瘤生长加速，导致某种治疗失败，形成一种治疗不敏感性交替发生的现象，们称之为肿瘤耐药性的漂移，这实际上是肿瘤基因多态性的一个表达结果，因此希望借助于有计划地选择这些曾经敏感的药物交替序贯应用治疗NSCLC以解决这一问题。

我们的前期研究完成了采用SELDI技术预测吉非替尼治疗NSCLC的疗效，以决定吉非替尼治疗NSCLC的取舍，在长达3年的研究中我们认为采用SELDI技术不仅可以发现吉非替尼治疗NSCLC的原发性耐药，也能发现继发性耐药。其蛋白质指纹图谱M/Z：8, 693±50H^+^丰度≤25%，可以作为吉非替尼治疗NSCLC患者取舍的筛选指标^[15, 16]^，因此，治疗期间M/Z：8, 693±50H^+^指纹丰度由小于25%发展至大于25%可认为是获得性耐药的标记指纹。很显然，肿瘤耐药性的漂移现象能被SELDI技术发现。因此根据SELDI指纹的不同标记有计划地应用含铂化疗方案和吉非替尼序贯治疗NSCLC，以解决含铂方案化疗失败后应用吉非替尼治疗失败后的再治疗问题。

在本研究的9例患者中，中位总生存期为27个月（10个月-66个月），有4例规律行化疗及吉非替尼序贯治疗，2例患者服吉非替尼期间出现肝转移，行化疗，疗效SD后继续口服吉非替尼。

另有3例患者，其中1例在服吉非替尼期间由于口鼻和颜面严重皮疹且在服药时发作而不得不停用吉非替尼，其蛋白质指纹检查M/Z: 8, 693±50H^+^丰度 < 10%。化疗效果差，化疗后4个月死亡，总生存期为21个月; 1例患者在服用吉非替尼7个月后因肿瘤增大行指纹检查发现耐药，化疗3周期，疗效PD，后由于轻微副反应及经济问题放弃治疗，总生存期达23个月; 另1例口服吉非替尼后由于耐药间断化疗2年，化疗期间未规律行SELDI检查，因肿瘤增大死亡，总生存期达25个月。

从以上结果可以看出，除2例不能入组外，其余都进行了两个循环周期的治疗，显然，吉非替尼对NSCLC的治疗发生耐药性的漂移是存在的，但从结果中又看到，生存期低于30个月的患者几乎都死于肿瘤复发和转移，显然针对吉非替尼耐药漂移的治疗，仅选择与含铂方案的序贯化疗是不足的，应根据肿块变化和肿瘤标记物的变化给予同步治疗是非常有必要的。

**1:** 9例晚期NSCLC患者治疗情况 The treatment records of nine patients with non-small cell lung cancer

Patients	Sequential number	TTD	The illness or cause of death
A	5	43	Cause of the death: severe lung infection, respiratory failure
B	2	27	Cause of the death: tumor recurrence
C	3	42	Cause of the death: cerebral haemorrhage
D	2	28	Cause of the death: brain metastasis
E	2	10	Cause of the death: brain metastasis, liver metastasis
F	3	66	Cause of the death: abdominal metastases, cachexia
G	1	21	Because of the severe rash to give up gefitinib, then accepted chemotherapy, and 4 months later, death, during the 4 months, intermittent took gefitinib, but at last gave up it due to the severe rash
H	2	23	Because of the side effects of chemotherapy and economic problems to give up chemotherapy, and death
I	3	25	Tumor increased before the death

我们认为：根据研究的结果，由于基因多态性导致有一部分患者出现耐药性的漂移，使常规治疗失败，同时，这种针对NSCLC患者抗肿瘤药物耐药的漂移现象表明含铂方案和吉非替尼之间存在有抗NSCLC作用的互补性，其互补性的基础是如何能发现并预测优势肿瘤和劣势肿瘤。SELDI技术可以通过指纹标记发现并预测这些现象，以此做出诊断，以SELDI标记指纹作为含铂方案化疗和吉非替尼治疗NSCLC的取舍标记，有可能提高NSCLC患者药物治疗的获益率和获益时间，以此延长生存期。这种序贯治疗选择的取舍是在借助于SELDI指纹对某种药物治疗疗效预测的平台上完成的，是以预测结果作为应用药物治疗取、舍的标记，更能体现早诊、早治的肿瘤治疗理念，因此，根据前瞻性研究的结论，有必要扩大研究范围进行深入研究。
